# In Memoriam: Prof. Dr. Giovanni Ralli

**DOI:** 10.3390/audiolres16040100

**Published:** 2026-07-03

**Authors:** Giacinto Asprella-Libonati

**Affiliations:** Otolaryngology Unit, Madonna Delle Grazie Hospital of Matera, 75100 Matera, Italy; giacintoasprella@gmail.com

With profound sorrow, I announce the passing of Prof. Dr. Giovanni Ralli, who passed away last night.

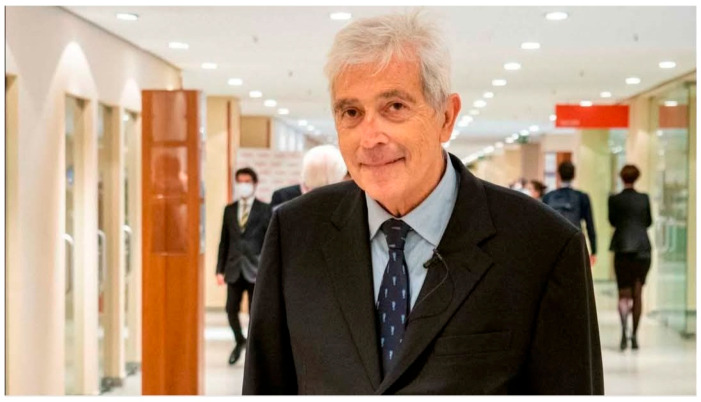



Prof. Ralli was a distinguished figure in Italian otorhinolaryngology, audiology, otology, and vestibology. He served for many years at the Department of Sense Organs, Sapienza University of Rome, contributing with dedication to clinical practice, academic teaching, and scientific research. He was also a valued member of the Editorial Board of *Audiology Research*, where his experience, authority, and human qualities enriched the scientific community of our journal.

His academic and professional path was deeply linked to the Roman School of otorhinolaryngology and vestibology. Through his teaching, clinical activity, and scientific contributions, Prof. Ralli helped shape generations of physicians, audiologists, vestibologists, and researchers. His work reflected a rare balance between rigorous clinical observation, scientific curiosity, and sincere attention to patients.

For many of us, however, Giovanni was much more than a professor, a clinician, or a colleague. He was a friend, a mentor, and a man of extraordinary kindness. Personally, I lose not only an esteemed member of the *Audiology Research* family, but a fraternal friend. His presence, his advice, his culture, and his generous humanity accompanied many important moments of my professional and personal life.

Prof. Ralli’s scientific interests included ENT, otology, audiology, and vestibular disorders, fields to which he contributed through publications, academic teaching, and collaborative work.

Only recently, he contributed to the historical and scientific memory of our discipline, including work devoted to the contribution of the Roman School to vestibology, a topic that well represents his role as a bridge between tradition, clinical experience, and future generations.

On behalf of *Audiology Research*, its Editorial Board, authors, reviewers, and readers, I extend our deepest condolences to his family, his colleagues, his students, and all those who had the privilege of knowing him.

We will remember Prof. Giovanni Ralli with gratitude, affection, and respect. His legacy will remain alive in the people he taught, in the patients he cared for, in the colleagues he inspired, and in the scientific community he helped to build.

Farewell, dear Giovanni.

Your voice, your wisdom, and your friendship will remain with us.

